# The Use of Keystone Flaps in Periarticular Wound Closure: A Case Series

**DOI:** 10.3389/fsurg.2017.00068

**Published:** 2017-11-29

**Authors:** Thomas H. Jovic, Zita M. Jessop, Robert Slade, Thomas Dobbs, Iain S. Whitaker

**Affiliations:** ^1^Welsh Centre for Burns and Plastic Surgery, Morriston Hospital, Swansea, United Kingdom; ^2^Reconstructive Surgery and Regenerative Medicine Research Group, Institute of Life Sciences, Swansea, United Kingdom

**Keywords:** periarticular, wound closure, skin cancer, fasciocutaneous flaps, keystone flaps

## Abstract

The Keystone perforator island flap (Keystone flap), is a Type A fasciocutaneous advancement flap, consisting of two V to Y advancement flaps. Skin cancer excision around joints presents a number of reconstructive challenges. Owing to the mobile nature of joints, the optimal periarticular reconstructive option should possess the ability to provide adequate tissue coverage and withstand regional changes in tensile pressures. We report a single-surgeon series of five cases of periarticular keystone flap between 2014 and 2017. Data were collected from operation notes, clinical photography, histopathology, and outpatient clinic records. The indication for keystone flap was skin cancer in all cases (*n* = 5). The largest defect size post-excision in was 75 mm × 40 mm × 15 mm. All keystone flaps demonstrate a color and cosmetic appearance comparable to adjacent tissue. There were no major postoperative complications including flap failure or impaired range of joint movement in the follow up period. Superficial wound infection occurred postoperatively in one case. This is the first case series to discuss the use of keystone flaps in periarticular wound closure. Locoregional fasciocutaneous wound coverage offered by keystone flaps may alleviate the risks of graft failure, contour defects, and donor site morbidity associated with alternative reconstructive options, with good functional and cosmetic outcomes. We advocate their use as a robust reconstructive option in periarticular areas.

## Introduction

We report a single-surgeon series of five cases of periarticular keystone flap between 2014 and 2017 (Table 1). Data were collected from operation notes, clinical photography, histopathology, and outpatient clinic records. All patients provided written informed consent for their images and data to be used for research and publication.

**Table 1:** Summary of lesion type, size, post-excision defect size, keystone flap subtype, and postoperative complications for included cases.

Case	Age (gender)	Lesion	Lesion size (mm)	Area	Associated joint(s)	Defect size (mm)			Histology	Keystone flap	Follow-up period (months)
						Length	Width	Depth			
1	63 (M)	Nodular BCC	18 × 18	Left shoulder	Glenohumeral	70	35	5	Clear with 3 mm margins	Type 2a	4
2	79 (F)	Melanoma scar	32 × 22	Left popliteal fossa	Knee	55	40	12	No residual melanoma	Type 3	18
3	71 (F)	Melanoma scar	45 × 1	Left forearm	Elbow	75	40	17	No residual melanoma	Type 3	10
4	58 (F)	Melanoma scar	9 × 8	Left medial malleolus	Ankle	27	12	2	No residual melanoma	Type 1	3
5	65 (M)	Nodular BCC	27 × 20	Left shoulder	Glenohumeral	35	56	11	Nearest margin 3.9 mm	Type 1	3

The indication for keystone flap was skin cancer in all cases (*n* = 5), consisting of two basal cell carcinomas (BCC) and three melanoma scar excisions. All cases were performed as day case operations. In all cases, melanomas were initially excised with 2 mm margins to determine Breslow thickness, and the subsequent scar revised with appropriate margins prior to reconstruction. BCC were excised with 4–5 mm margins as per British Association of Dermatology guidelines (1) and demonstrated adequately clear excision margins histologically (Table 1).

The defect size post-excision ranged from 75 mm × 40 mm × 17 mm to 27 mm × 12 mm × 2 mm with a mean size of 52 mm × 37 mm × 9 mm (Table 1). Two keystone flaps were Type 1, two Type 3 flaps (popliteal and antecubital fossa), and a Type 2a flap adjacent to the glenohumeral joint (Table 1).

**Figure 1:**
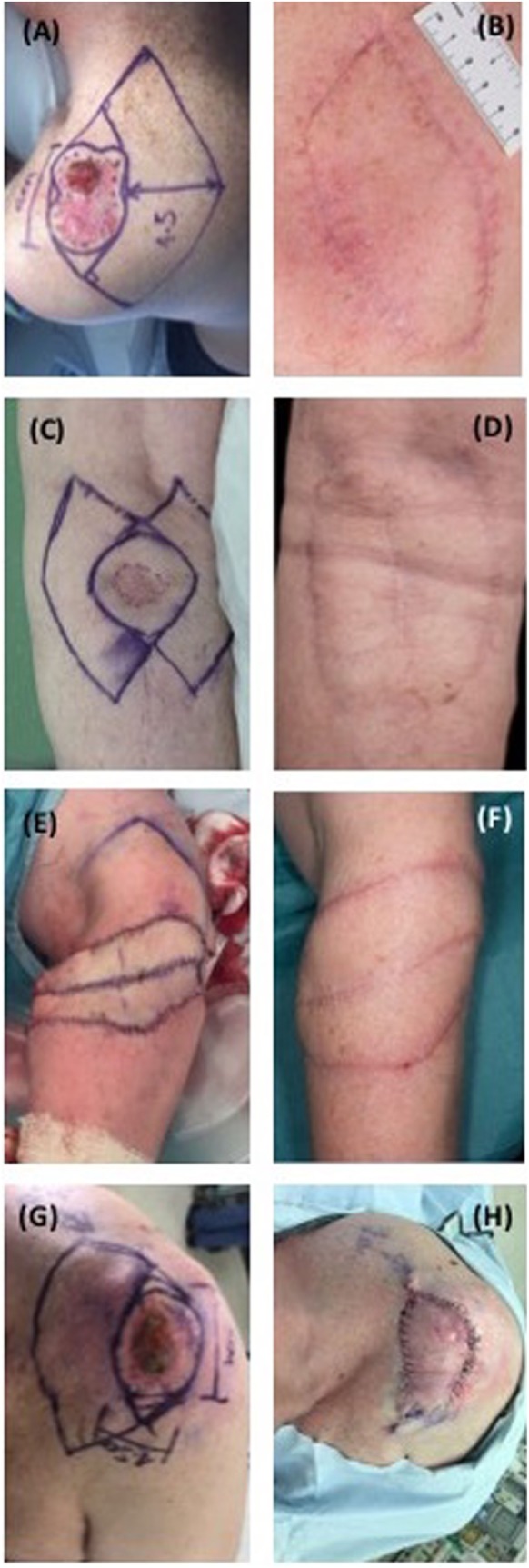
Preoperative, intra-operative and postoperative periarticular keystone flaps. **(A)** Patient 1, pre-operative; **(B)** patient 1, 2 months postoperative; **(C)** patient 2, pre-operative; **(D)** patient 2, 2 months postoperative; **(E)** patient 3, intra-operative; **(F)** patient 3, 1 month postoperative; **(G)** patient 5, pre-operative; **(H)** patient 5, intra-operative.

All keystone flaps demonstrated a color and cosmetic appearance comparable to adjacent tissue, with minimal scar formation at the periphery (Figure 1). Impaired range of joint movement was neither reported subjectively nor detectable on clinical examination, plus no issues regarding contracture or problematic scarring have been identified in this series to date during the follow-up period. One patient was readmitted 7 days postoperatively with erythema and swelling underlying the keystone flap (Case 5) consistent with a superficial wound infection. Blood tests at the time of readmission demonstrated a normal white cell count and mildly elevated C-reactive protein. Methicillin-sensitive *Staphylococcus aureus* was identified from wound swabs to be the causative organism. The infection was successfully treated with 7 days of clarithromycin, with no adverse impact on the subsequent healing process. All patients have reported high levels of satisfaction with the appearance of their reconstruction.

## Background

The Keystone perforator island flap (Keystone flap), is a local, Type A fasciocutaneous advancement flap, consisting of two V to Y advancement flaps (2). Originally described by Behan et al. (2), its use as a locoregional reconstructive tool offers an attractive alternative to skin grafting and free tissue transfer, reducing complications associated with donor site harvest, blood supply, and cosmetic donor–recipient mismatches (3).

Blood supply to the flap is based on random vascular perforators, with a dual supply from both the subcutaneous vascular plexus and perforating vessels in the fascial and muscular layers (4).

Since their introduction, keystone flaps have been subdivided into four subtypes (Table 1) and used for head and neck (5–8) and lower limb reconstruction (9–11), to resurface irradiated tissue or burn excisions (12, 13) and in mobile areas such as the lumbosacral spine (14, 15).

Periarticular wound closure is a unique reconstructive challenge due to multi-vector tensional forces (16). Adjacent tissue is continually subjected to stretch, compression and torsion and the reconstructive solution should possess the ability to withstand changes in tensile forces. It is widely known that periarticular skin grafts are subjected to movement and shearing forces, disrupting the formation of early fibrin bonds and leading to increased failure rates (16–18) and contracture (19, 20). In our experience, the use of locoregional flaps to reconstruct periarticular defects left from skin cancer excisions are superior to skin grafts, and we illustrate this through the use of the keystone flap in five patients.

## Discussion

To our knowledge, this is the first case series to discuss the use of keystone flaps in periarticular wound closure. Our experience highlights a number of advantages and important learning points to consider during patient selection.

In our study of five patients, the cosmetic outcomes were well-received and patient satisfaction was high. This parallels studies using keystone flaps in esthetically sensitive areas such as nasal and facial reconstruction (5–7, 21–23). Despite their cosmetic appeal in matching the appearance of adjacent skin (3) a notable scar burden may still be associated with the use of these flaps, especially when under high tension, although this ameliorates with time (Figure 1).

Periarticular wound closure can prove challenging. Moreover, the large antecubital and popliteal fossa defects (Table 2) presented a range of reconstructive challenges beyond the issues of multi-vector tensile forces, such as exposed neurovascular structures needing robust soft tissue protective cover. The burden of skin cancers in mobile areas, such as the lower leg is high, especially in females (24), and to achieve clear surgical margins often creates large defects. Despite this, there is a sparsity of literature that concerns itself with the most appropriate means of periarticular wound closure. Although keystone flaps have been used previously for generic lower limb reconstruction (6, 25) they have not been used widely in the popliteal fossa or periarticular areas to our knowledge. Alternatives to fasciocutaneous flaps such as the keystone include reconstruction with skin grafts, though these do not parallel the robust tissue coverage of local flaps and may exhibit high failure rates (16), particularly in mobile areas where graft adherence may be compromised. In addition, contour defects, pigmentation mismatch (26), and secondary contractures (19) may also restrict cosmetic and functional outcomes, particularly when extrapolated to periarticular defects. Alternative flaps for lower limb wound closure may include reverse flow flaps and local transposition flaps (27). Reverse flow flaps may compromise the arterial inflow to the foot (27), and although comparable wound coverage may be achieved through transposition, defects of large size or low tissue laxity may require concomitant skin grafting to close the donor site. Similarly, pedicled fasciocutaneous flaps, such as the radial forearm flap (28) have been documented in upper limb periarticular wound closure. However, this often requires a skin graft to close the donor site, and division of the arterial inflow to the hand at the expense of wound closure (29).

**Table 2:** Subtypes of Keystone Flap and their surgical applications [Modified from Behan et al 2003 (1); Pelissier et al 2007 (3)].

Keystone flap subtype	Principles and surgical applications
Type I	Primary defect less than 2 cm width
	Lateral deep fascia remains intact

Type IIa	Defects greater than 2 cm
	Division of deep fascia required to facilitate tissue mobilization

Type IIb	Useful in large defect coverage
	Concomitant use of split-skin graft, reduces tension on flap margins

Type III	Large primary defect (5–10 cm)
	Two keystone flaps on each border of the defect

Type IV	Rotational keystone flap, useful in joint contracture or open fractures
	Flap is raised with up to 50% sub-facial undermining

It has been reported that keystone flaps around the elbow, knee, and ankle joints should be used cautiously due to reduced skin laxity and a risk of dehiscence (30), and previous studies have reported wound dehiscence following full flexion at the lumbosacral area (25). In our series, we did not immobilize the joints in plaster casts, but advise bulky dressings and enforced rest until wound checks were performed 2 weeks postoperatively. We present several examples of successful keystone flap reconstruction around the joints of the upper and lower limbs with no major complications and throughout the postoperative follow-up, both patient and clinicians were satisfied with the quality of the functional and cosmetic results.

## Concluding Remarks

Keystone flaps provide an effective means of periarticular wound closure in an area of high mobility and low skin laxity. Locoregional fasciocutaneous wound coverage offered by keystone flaps provide a good cosmetic match, robust soft tissue coverage and avoid contour defects and contracture at a cost of minimal donor site morbidity. On the basis of our experience, we advocate the increased use of the keystone flap to close periarticular defects following skin cancer excision.

## Ethics Statement

This is a retrospective, descriptive study in which all patients provided written consent for their images to be used for publication purposes.

## Author Contributions

All authors (TJ, ZJ, RS, TD, and IW) have made substantial contributions to the conception or design of the work; or the acquisition, analysis, or interpretation of data for the work. All authors have been involved in drafting the work or revising it critically for important intellectual content and approve the final version to be published. All authors agree to be accountable for all aspects of the work in ensuring that questions related to the accuracy or integrity of any part of the work are appropriately investigated and resolved.

## Conflict of Interest Statement

The authors declare that the research was conducted in the absence of any commercial or financial relationships that could be construed as a potential conflict of interest.
